# UMAI-WINGS: Evaluating the Effectiveness of Implementing mHealth Intimate Partner Violence Prevention Intervention in Reducing Intimate Partner Violence Among Women from Key Affected Populations in Kazakhstan Using a Community-Based Approach

**DOI:** 10.3390/bs15050641

**Published:** 2025-05-09

**Authors:** Assel Terlikbayeva, Sholpan Primbetova, Ohshue S. Gatanaga, Mingway Chang, Yelena Rozental, Meruert Nurkatova, Zulfiya Baisakova, Yelena Bilokon, Shelly E. Karan, Anindita Dasgupta, Louisa Gilbert

**Affiliations:** 1 Center of Scientific and Practical Initiatives, 38B, Shashkina Str., Almaty 050040, Kazakhstan; assel.terlikbayeva@ghrcca.org (A.T.); elena.rozental@ghrcca.org (Y.R.); mira.nurkatova@ghrcca.org (M.N.); 2 School of Social Work, Columbia University, New York, NY 10027, USA; mpc2004@columbia.edu (M.C.); sek2233@columbia.edu (S.E.K.); lg123@columbia.edu (L.G.); 3 Global Health Research Center of Central Asia, Almaty 050040, Kazakhstan; 4 Department of Sociology and Social Work, Faculty of Philosophy and Political Science, Al-Farabi Kazakh National University, Almaty 050040, Kazakhstan; 5 Department of Health Systems and Population Health, School of Public Health, University of Washington, Seattle, WA 98195, USA; ohshue@uw.edu; 6 Social Intervention Group, School of Social Work, Columbia University, New York, NY 10027, USA; 7 Crisis Center Union, Almaty 050012, Kazakhstan; kcalmaty2017@gmail.com; 8 Public Foundation “Revansh”, Almaty 050038, Kazakhstan; bilokon-21@mail.ru; 9 Department of Sociomedical Sciences, Mailman School of Public Health, Columbia University, New York, NY 10032, USA; ad3341@columbia.edu

**Keywords:** intimate partner violence, gender-based violence, key populations, Kazakhstan, community coalitions

## Abstract

Key affected populations (KAPs), including women who use drugs, engage in sex work, or live with HIV, are disproportionately affected by HIV, gender-based violence, substance use, and mental health. In Kazakhstan, they face significantly higher rates of intimate partner violence (IPV), with prevalence ranging from 45% to 75% compared to the national average of 27%, alongside barriers to accessing IPV services. This community-level implementation trial with a waitlist control group evaluated the effectiveness, safety, and acceptability of a self-paced mobile health intervention (UMAI-WINGS) for women from KAPs in Kazakhstan. The study enrolled 508 women, with 306 in Almaty City (intervention) and 200 in Almaty Oblast (waitlist control). IPV rates (physical, sexual, and psychological) were assessed at baseline and 6-months post-intervention. Participants in the intervention community were significantly less likely to report psychological (−23.0%), sexual (−27.0%), and physical IPV (−29.0%) at the 6-month follow-up compared to the control group. These results demonstrate the potential of digital, community-driven interventions to reduce IPV among marginalized women and offer a scalable, evidence-based model for implementation. The success of the UMAI-WINGS underscores the value of community-based, mobile health approaches for advancing trauma-informed, survivor-centered care and closing critical service gaps for underserved populations.

## 1. Introduction

### 1.1. Global and National Overview of IPV

Globally, 1 in 3 women experience physical and/or sexual intimate partner violence (IPV) or non-partner sexual violence in their lifetime (WHO, 2021). In Kazakhstan, 27% of women reported IPV experiences in 2023 (World Bank, 2024). A 2019–2022 national survey of 14,342 women who sought forensic support found that 78% had experienced physical violence, 21% psychological violence, 16% sexual and physical violence, and 6% regular sexual violence (Mussabekova et al., 2024). In 2024, more than 100,000 domestic violence cases were reported, resulting in 72,000 protection orders and 16,000 court decisions (ORDA, 2024).

### 1.2. IPV and Risk Factors in Key Affected Populations (KAPs)

KAPs in this study include women who use drugs, engage in sex work, live with HIV/AIDS, or identify as transgender. These women experience significantly higher rates of IPV than the general population. A meta-analysis found IPV prevalence among KAPs ranges from 23% to 75%, often due to criminalization, stigma, and exclusion from national surveys (El-Bassel et al., 2022). Transgender women, in particular, face a 1.7 times higher risk of any IPV and 2.5 times higher risk of sexual IPV than cisgender individuals (Peitzmeier et al., 2020).

In Kazakhstan, one study found that 45% of women who exchanged sex for money or drugs experienced IPV, and 28% experienced violence from non-intimate partners in the past 90 days (Mukherjee et al., 2023). Another study reported that 52% of HIV-positive women experienced lifetime IPV (Jiwatram-Negrón et al., 2018). No IPV prevalence data exist for transgender women in Kazakhstan, underscoring a critical gap in research.

### 1.3. Multiple Risk Factors Drive IPV Among KAPs

These include low educational attainment, early marriage, childhood exposure to violence, substance use, and mental health issues such as depression (L. J. Brown et al., 2023; Gunarathne et al., 2023; Sabri & Young, 2022). Relationship stressors, like economic hardship and male unemployment, further increase risk (Murphy et al., 2021). Harmful gender norms, such as the acceptance of male dominance and justification of violence against women, remain prevalent in Kazakhstan, where over 93% of citizens hold gender-biased views (Senem Sel, 2024).

### 1.4. Structural Barriers in Kazakhstan

Although a new law was passed on 15 April 2024 criminalizing domestic violence, significant service and policy gaps persist (Human Rights Watch, 2024). Most crisis centers—despite recent expansion—lack resources, are not inclusive, and often fail to admit women from KAPs, particularly those living with HIV, using drugs, or identifying as transgender (United Nations Development Programme, n.d.; CABAR.Asia, n.d.).

Four major barriers limit effective IPV response for KAPs in Kazakhstan:

(1) Lack of surveillance data: national household surveys exclude KAPs, preventing the timely identification of IPV hotspots;
(2) Fragmented services: there is little coordination between mainstream IPV services and programs serving women from KAPs;
(3) Stigma and discrimination: widespread blame of KAPs for the violence they experience inhibits access to support;
(4) Limited community engagement: few mechanisms exist to mobilize stakeholders for inclusive policy and service development.

### 1.5. Study Rationale and Aim

This study addresses service gaps for marginalized women by evaluating the implementation and outcomes of UMAI-WINGS, a community-based mHealth intervention adapted from the evidence-based WINGS model (Gilbert et al., 2016, 2017), an IPV Screening, Brief Intervention, and Referral to Treatment (SBIRT) model, adapted for women from KAPs using a community-driven approach in Kazakhstan. WINGS has shown effectiveness among women who use drugs in the U.S. and Kyrgyzstan and is built on a screening, brief intervention, and referral to treatment (SBIRT) framework. UMAI-WINGS incorporates a multisectoral, community-coordinated response model tailored for KAPs in Kazakhstan. This trial assessed the acceptability, safety, and effectiveness of UMAI-WINGS in reducing IPV over six months among 458 women across Almaty City (intervention) and Almaty Oblast (control). It also examined whether outcomes varied by KAP subgroup.

## 2. Materials and Methods

### 2.1. Study Design

**Figure 1:**
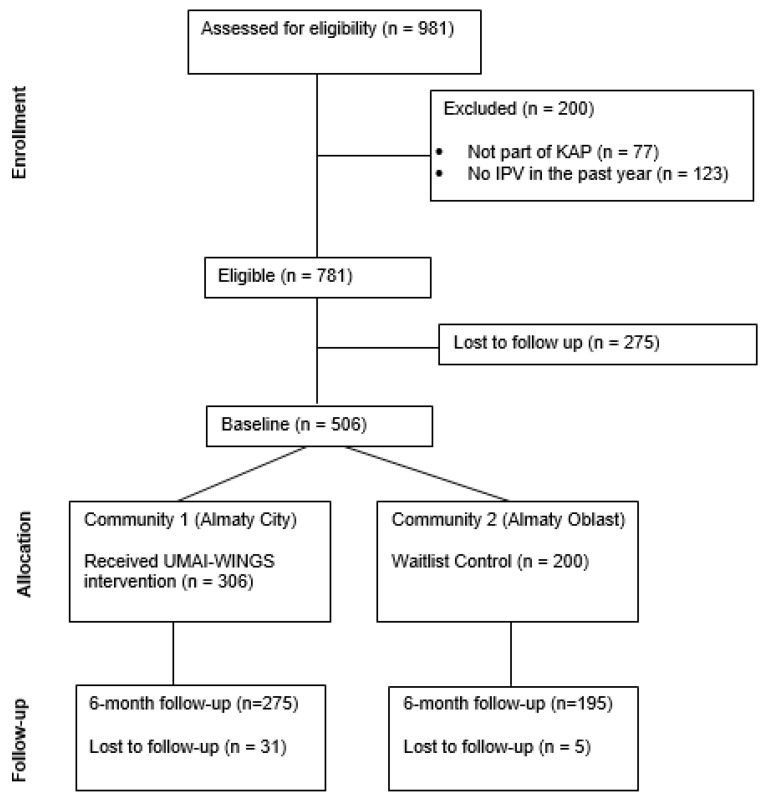
CONSORT diagram.

We opted for a community-level implementation trial, rather than an individual-level randomized controlled trial, to evaluate the effectiveness of a community-level engagement approach to implementing the UMAI-WINGS intervention and building a coordinated service network for women from KAPs through partner organizations. This design allowed us to assess not only individual-level outcomes but also how intervention delivery embedded within a community system influences access, safety, and linkage to care, which is particularly critical for marginalized women facing structural stigma. This waitlist control community trial was conducted between April 2022 and December 2024 in Almaty City (intervention community) and Almaty Oblast (control community) in Kazakhstan. Study participants completed quantitative assessments at the baseline and 6-month follow-up. After the 6-month follow-up, the waitlist control community also received the intervention. Pre–post outcomes were compared between the two communities to evaluate the intervention impact (see CONSORT diagram, Figure 1). The study was reviewed by the Al-Farabi Kazakh National University Ethics Committee (IRB00010790) and the Columbia University Institutional Review Board (IRB-AAAU4607).

### 2.2. Study Participants

This study included women who (1) were at least 18 years old and (2) identified as part of a KAP by meeting one or more of the following criteria: (a) exchanged sex for money, alcohol, drugs, or other goods/resources in the past year; (b) reported living with HIV/AIDS; (c) reported injecting or using drugs and/or binge drinking (defined as consuming 4+ drinks in one sitting) in the past year; (d) identified as a transgender woman; and (e) reported experiencing IPV in the past year. Women were excluded if they (1) had a cognitive or psychiatric impairment that would interfere with comprehension of study procedures (as assessed during informed consent); (2) did not speak and understand Russian at a conversational level; or (3) were unwilling or unable to complete informed consent or participate in the study.

Participants were recruited in person by trained outreach workers affiliated with community-based organizations and health care clinics serving KAPs in Almaty and Almaty Oblast. These included organizations such as Revansh, Community Friends, Transdocha, and Amelia, which have longstanding trust and engagement with KAP communities. Outreach workers conducted targeted outreach in community hotspots, clinics, and support centers. They identified potentially eligible participants, provided brief descriptions of the study, conducted preliminary screening, and obtained verbal agreement to be contacted by the study team. Outreach workers played a critical role in establishing trust, ensuring cultural and contextual sensitivity, and reducing barriers to participation for women who may otherwise be hesitant to engage in research due to stigma or safety concerns.

Women who met the eligibility criteria and agreed to participate were invited to the CBO offices to complete informed consent procedures. Upon enrollment, the participants completed a baseline survey via computer-assisted self-interviews (CASIs). Those in Almaty City (intervention community) immediately received access to the UMAI–WINGS self-guided mHealth intervention following baseline. Participants from both the intervention and waitlist control communities (Almaty Oblast) completed a 6-month follow-up assessment via a CASI. After completing follow-up, participants in the control group were provided access to the intervention.

### 2.3. Intervention: UMAI-WINGS Community Coordinated Response Model and Role of the Community Action and Accountability Board (CAAB)

The UMAI-WINGS intervention is a combined screening, brief intervention, and referral tool (SBIRT) implemented using a community-coordinated response (CCR) model to address IPV among women from KAPs. Guided by social cognitive theory, UMAI-WINGS was adapted from the evidence-based intervention WINGS (Gilbert et al., 2015b), which has been successfully implemented in multiple low-resource countries, such as Kyrgyzstan, India, and Ukraine.

UMAI-WINGS SBIRT’s core components were adapted from the original WINGS 7 core components: (1) raising awareness about different types of IPV and risk factors (i.e., substance abuse) for IPV; (2) screening to identify different types of IPV in women and provide individualized feedback (none, some, or high risk); (3) eliciting motivation to address IPV and relationship conflict, safety planning using motivational interviewing; (4) conducting safety planning to reduce risks off exposure to IPV; (5) enhancing social support to address relationship conflict and IPV; (6) setting goals to improve relationship safety and reduce risks of IPV; and (7) identifying and prioritizing service needs, linkage to IPV, and other services.

The UMAI-WINGS intervention was adapted through an iterative, community-informed process involving members of the Community Action and Accountability Boards (CAABs), partner NGOs, and women from KAPs. Specific cultural adaptations included revising content to reflect locally relevant IPV scenarios, rephrasing safety planning prompts using language appropriate for Russian and Kazakh speakers, and incorporating guidance on navigating service discrimination frequently faced by sex workers, transgender women, and women living with HIV. Policy-related adaptations included referral linkages to local harm reduction, HIV, and legal aid services, as well as guidance for women with prior incarceration or drug use history, populations that are often excluded from mainstream IPV shelters in Kazakhstan.

The UMAI-WINGS intervention was delivered by staff at partner organizations that serve women from KAPs. This mHealth tool was adjusted to be accessible via mobile devices, allowing women to engage with IPV-related education, safety planning, and support resources at their own convenience and without requiring face-to-face interactions. This digital approach enhances privacy, reduces logistical barriers, and ensures scalability for reaching marginalized women who might otherwise face challenges in accessing traditional IPV services. Nevertheless, some participants preferred to go through the intervention at our partner organizations. To ensure the participant’s safety while using the tool, the participants were instructed to complete the intervention alone in a private space and to exit the tool using a “safe and save exit button” if they were interrupted. The participants were given options about how to receive their safety plan and service referrals (email, mail, or in person).

To adapt and implement UMAI-WINGS within the Kazakhstan context, the project engaged a Community Action and Accountability Board (CAAB) in each site (Almaty City and Almaty Oblast). Each CAAB was composed of local IPV/GBV service providers, law enforcement, NGO representatives serving women from KAPs, IPV/GBV advocates, and women with lived experience of IPV/GBV from KAPs. The CAABs played a critical role in localizing the intervention, ensuring cultural and structural relevance, building multi-sectoral trust, and enhancing the long-term sustainability of the intervention ecosystem.

The CAAB’s engagement included the following key activities:

(1) Developing a shared charter: at the outset, CAAB members collaboratively created a shared vision and operating agreement to guide their work in addressing IPV/GBV in the community.
(2) Reviewing local IPV/GBV data: the CAAB reviewed existing data on the prevalence and dynamics of IPV/GBV among KAPs in their communities, fostering shared understanding of the magnitude and complexity of the problem.
(3) Service mapping and gap analysis: CAAB members conducted a comprehensive mapping of local IPV/GBV-related services, identified service gaps (e.g., lack of inclusive shelters, legal aid, or mental health care), and pinpointed structural barriers (e.g., stigma, discrimination, exclusionary policies, and lack of transportation) that prevent KAP women from accessing care.
(4) Network building across sectors: the CAAB facilitated the development of a cross-sectoral network to enhance service coordination, streamline referrals, and strengthen the safety net for women experiencing IPV.
(5) Capacity building and training: CAAB members were trained on the UMAI-WINGS intervention content and implementation procedures; trainings also covered how to interpret and use UMAI-WINGS data for planning and advocacy, promoting evidence-informed local responses to IPV/GBV.
(6) Participatory adaptation: CAAB feedback was central to adapting the original WINGS model; CAABs and KAP representatives informed ethical adjustments, content refinement, and cultural tailoring, especially regarding safety planning components for women engaged in sex work and transgender women, where the highest risk and greatest barriers were identified.

The CAAB was crucial not only for intervention fidelity but also for community ownership, trust building, and long-term sustainability. Their involvement ensured the intervention addressed real-life barriers, reflected the lived experiences of women from KAPs, and built bridges between formal systems (e.g., shelters and clinics) and community-based actors, making the model scalable across similar high-stigma, low-resource settings.

For this project, we employed an adaptation process entailing: (1) eliciting feedback on the content and activities of the original WINGS and CTC intervention from representatives of KAPs and CAAB members and feedback on ethical considerations and the local context from our CAABs in two communities and partner service NGOs (especially with transgender NGOs); (2) making refinements to the intervention based on this feedback; (3) safety planning for WSW and TGW required most formative research and adaptation; (4) identifying barriers and facilitators of implementing the UMAI + CCR interventions with partner organizations and developing an intervention protocol; (5) piloting the adapted UMAI-WINGS intervention with 5 women from targeted groups and revising intervention based on their feedback; and (6) developing the final version of the UMAI-WINGS intervention for implementation.

### 2.4. Community Engagement and Capacity Building in Intervention Trial

A community engagement approach was embedded throughout the process of working with CAABs and focused on co-learning, capacity building, and relationship building. To build the capacity of community service providers and mainstream GBV shelters on GBV identification, screening, safety planning, and linking to services, a series of trainings on the UMAI-WINGS intervention and CCR model were conducted: (1) a standard 8 h training session based on the WINGS implementation manual; (2) stigma reduction and sensitivity training on working with key populations was conducted for police, medical, and social services providers (2023); and (3) training on trauma-based approaches when working with female survivors of IPV/GBV was conducted for IPV/GBV providers from crisis shelters). Community engagement was operationalized through the formation and continuous involvement of a Community Action and Accountability Board (CAAB) in each community, composed of representatives from IPV/GBV service organizations, police, NGOs serving key populations, and women with lived experience of IPV. Engagement mechanisms included collaborative development of a shared charter, service mapping and gap analysis, training on trauma-informed care, and joint implementation planning. Regular CAAB meetings created a platform for feedback loops and adaptive implementation strategies.

### 2.5. Measurement

Primary Outcomes: Intimate partner violence was measured with a shortened 8-item version of the Revised Conflict Tactics Scale (CTS2) (Gilbert et al., 2016; Straus et al., 1996), which assesses any severe/moderate sexual and physical violence by intimate partners (i.e., spouse, boyfriend/girlfriend, or regular sexual partners) in the past 6 months. These eight items include all the original CTS2 items that assess severe/moderate sexual and physical IPV with dichotomous yes/no responses. For example, the two items that assess the experience of severe physical IPV by male partners included the following: (1) Has a male/female partner(s) ever kicked you, slammed you against a wall, beaten you up, punched or kicked you, hit you with something that could hurt or burned or scalded you on purpose? (2) Has a male/female ever choked you or used or threatened to use a knife or gun on you? If the participants responded yes to either question, they were coded as positive for experiencing lifetime severe physical violence from male/female partners. Psychological IPV was assessed with two psychological abuse items from the WAST screening tool (J. B. Brown et al., 2000): (1) have you been humiliated or emotionally abused by your partner or ex-partner? and (2) have you ever been afraid of your partner or ex-partner? If a participant responded yes to either question, they were coded as positive for psychological IPV. For each type of IPV, we compared any exposure to IPV in the past 6 months to none for the regression analyses.

Sociodemographics: This included the age in years, ethnicity (Russian, Kazakh, or other), less than high school education, household income, food insecurity in the past year (yes/no), marital status (married vs. not married), and housing insecurity in the past year (yes/no). In addition, we asked the following dichotomous items to assess the participant’s key affected population status: (1) Did you provide sexual services in exchange for money, alcohol, drugs, or any goods in the past 12 months? (yes/no); (2) Did you use any illicit drugs in the past 12 months? (yes/no); (3) Have you ever been diagnosed with HIV? (yes/no); (4) Do you consider yourself a transgender woman? (yes/no).

Acceptability and Safety: Participants were asked how satisfied they were with the UMAI program (satisfied, neither satisfied nor dissatisfied, or not satisfied), whether or not they used the safety plan, and whether or not they would recommend the UMAI-WINGS program at the 6-month follow-up. To assess the safety of participants, we trained research staff, outreach workers, and program staff at study partner organizations to be able to identify and report a wide range of negative incidents that may occur as a result of participating in the study and the UMAI-WINGS intervention. The investigative team assessed any negative incidents reported by staff to determine whether the incident occurred as a result of study participation, in which case it would be deemed an adverse event.

### 2.6. Data Analysis

We first computed descriptive statistics to summarize sociodemographic characteristics, key affected population (KAP) status, and IPV outcome measures at baseline and 6-month follow-up, both for the total sample and stratified by intervention (Almaty City) and waitlist control (Almaty Oblast) communities. To assess the differences between the intervention and control groups, we used two-tailed independent samples t-tests for continuous variables and Pearson’s χ^2^ tests for categorical variables. For IPV outcome measures at baseline and follow-up, we examined unadjusted risk ratios (RRs) using cross-tabulations to describe initial group differences.

To examine whether the effectiveness of the UMAI-WINGS intervention varied across KAP subgroups, we conducted moderation analyses using log-binomial regression models with interaction terms. Specifically, for each KAP subgroup (i.e., women who exchange sex for money or drugs, women living with HIV/AIDS, and women who use drugs), we fitted separate models that included the product term between study conditions (intervention vs. control) and the KAP subgroup indicator variable (e.g., intervention × sex work status). Each model also included the main effects of the intervention condition and the respective KAP subgroup, along with the same covariates used in the primary outcome models: baseline IPV outcome, HIV status, sex work engagement, drug use, ethnicity, marital status, history of homelessness, and past-year food insecurity. A significant interaction term was interpreted as evidence that the intervention effect was moderated by the KAP status, indicating differential effectiveness among subgroups. Although the UMAI-WINGS intervention was adapted to meet the needs of transgender women, the sample size (*n* = 24) was too small to conduct separate moderator analyses for this group. Nonetheless, transgender women were included in both the bivariate and multivariate outcome analyses.

All analyses were conducted using SAS version 9.4 (SAS Institute Inc., Cary, NC, USA). A two-tailed alpha level of 0.05 was used to determine statistical significance.

## 3. Results

### 3.1. Sample Flow and Retention

The participant flow is outlined in the CONSORT diagram (Figure 1). The UMAI-WINGS trial was conducted from April 2022 to December 2024 at two sites: Almaty City (intervention community) and Almaty Oblast (waitlist control). Of the 981 women screened, 781 met the eligibility criteria. Ineligible women (*n* = 200) were excluded for not belonging to a key affected population group (*n* = 77) or not reporting any IPV in the past year *(n* = 123). A total of 506 women enrolled and completed the baseline assessment (306 in the intervention community and 200 in the control community). At the 6-month follow-up, 470 participants (275 intervention and 195 control) completed the outcome assessment, reflecting a high retention rate of approximately 93%. The analytic sample for outcomes included 458 women who completed both the baseline and 6-month follow-up assessments after excluding 12 participants due to missing data.

### 3.2. Sociodemographic Characteristics

Baseline participant characteristics are presented in Table 1. The mean age was 36.7 years (SD = 9.0). Over one-third of participants were Kazakh (*n* = 169, 36.9%), about one-third were Russian (142 women, 31.0%), and the remainder identified as other ethnicities (147 women, 32.1%). Educational attainment was low: 101 participants (22.1%) had a 9th grade education or less, 145 (31.7%) had some secondary education, 161 (35.2%) completed high school and vocational training, and 51 (11.1%) held a Bachelor’s degree. One in ten women (*n* = 46, 10.0%) were currently married, indicating the vast majority were not married. Nearly two in five participants (186 women, 40.6%) had experienced homelessness in the past year, and over half (254 women, 55.5%) reported food insecurity in the past year. Almost three quarters (73.1%, *n* = 335) indicated that they exchanged sex for money or drugs in the past 12 months, 58.1% (*n* = 286) reported using drugs, 30.6% (*n* = 140) indicated that were living with HIV, and 5% (*n* = 24) identified as transgender women. Among transgender participants, 92% reported exchanging sex for money, alcohol or drugs; 42% were as diagnosed with HIV; and 79% reported using drugs. Compared to the waitlist control community participants, the intervention community participants were more likely to identify as Russian and less likely to experience food or housing insecurity. Intervention community participants were also more likely to report living with HIV but less likely to report using drugs or exchanging sex for money or drugs.

### 3.3. Baseline Group Comparisons

Table 2 presents descriptive statistics on IPV outcomes at each assessment and by intervention assignment. At baseline, there were several significant differences between the intervention and control community samples. The intervention community had a lower proportion of Kazakh participants (30.3% vs. 45.9%) and a higher proportion of Russian participants (39.4% vs. 19.6%) compared to the control community (*p* < 0.001). The intervention group also included a higher percentage of married women (12.9% vs. 6.2%, *p* = 0.019). Socioeconomic hardships were less common in the intervention community: past-year homelessness was reported by 31.1% of intervention participants vs. 53.6% of control participants and past-year food insecurity by 45.8% vs. 68.6% (both *p* < 0.001). The mean age and education levels did not differ significantly between the two groups (*p* > 0.05). Key population characteristics also differed by group. The intervention community had significantly fewer participants engaged in sex work (65.2% vs. 84.0%) and fewer who used drugs (45.5% vs. 75.3%) compared to the control community (*p* < 0.001 for both). Conversely, a greater proportion of women in the intervention community were living with HIV (40.2% vs. 17.5%, *p* < 0.001). There were no significant baseline differences in income or other assessed characteristics between the communities. A total of 64.6% (*n* = 296) reported experiencing psychological IPV in the past 6 months, 62.9% *(n* = 288) reported sexual IPV, and 58.3% (*n* = 267) reported physical IPV.

### 3.4. IPV Outcomes

Table 2 presents intimate partner violence outcomes at the baseline and 6-month follow-up for each group. At the baseline, IPV prevalence was high in both communities. Approximately two-thirds of participants had experienced psychological IPV in the past 6 months (64.0% in intervention vs. 65.5% in control) and a similar proportion had experienced sexual IPV (62.5% vs. 63.4%). Over half of participants reported physical IPV at the baseline (51.9% vs. 67.0%), with the intervention community showing a lower prevalence of physical IPV than the control community (this was the only significant baseline IPV difference). By the 6-month follow-up, IPV rates had declined substantially in the intervention community while remaining high in the control community. At 6 months, psychological IPV was reported by 41.7% of intervention participants versus 75.3% of control participants; sexual IPV by 43.6% vs. 70.6%; and physical IPV by 25.8% vs. 73.7% (intervention vs. control, all *p* < 0.001). These post-intervention differences indicate markedly lower IPV exposure in the intervention group compared to the control group at follow-up.

Table 3 presents the multivariable analyses of intimate partner violence outcomes after adjusting for IPV outcome measures at the baseline, sex worker status, HIV diagnosis, drug use, ethnicity, marital status, past-year homelessness, and past-year food insecurity. Compared to control community participants, intervention community participants were 23.0% less likely to report psychological IPV (aRR = 0.77 CI = 0.69, 0.86), 27% less likely to report sexual IPV (aRR = 0.73, CI = 0.63, 0.85), and 29% less likely to report physical IPV (aRR = 0.71, CI = 0.63. 0.80) at the 6-month follow-up.

None of these significant intervention effects for different types of IPV were moderated by any KAP subgroup (see Table 4 for moderator analyses).

### 3.5. Acceptability

At the 6-month follow-up, participants in the intervention community reported high acceptability of the UMAI-WINGS program. Among those who completed the follow-up (*n* = 275), 223 women (81.1%) were satisfied with the program, 38 (13.8%) were neither satisfied nor dissatisfied, and 14 (5.1%) were not satisfied. Nearly all participants (259 women, 94.2%) said they would recommend the UMAI-WINGS intervention to other women. Over half of the participants reported using the safety plan provided, and 96% found the safety-planning activity to be useful.

### 3.6. Safety

No significant safety issues arose during the study. Research and program staff did not report any negative incidents related to study participation, indicating that no adverse events occurred as a result of the intervention.

## 4. Discussion

The study findings indicate that women in the UMAI-WINGS IPV SBIRT intervention community were significantly more likely to reduce their experiences of all types of IPV compared to women in the control community in Kazakhstan, consistent with prior studies documenting the efficacy of this IPV SBIRT intervention with women from KAPs (Gilbert et al., 2016; Gilbert et al., 2017). The lack of moderator effects by subgroups (i.e., WUD, WSW, and WLH) further suggests that the intervention was effective for all KAPs. To date, this is the largest community-based trial of an IPV SBIRT intervention with key affected populations of women.

Although the intervention was adapted for transgender women and we successfully enrolled 24 participants, the small sample size precluded us from conducting subgroup analyses. Future studies should intentionally oversample transgender women and other under-represented KAPs to ensure that the findings are inclusive and allow for tailored intervention refinement. Moreover, this study found that the UMAI-WINGS intervention was acceptable to study participants, as indicated by a high satisfaction rating, and safe, as indicated by the absence of adverse events.

This trial employed innovative multi-level implementation strategies to address community-level and organizational barriers that prevent women from KAPs from accessing IPV services and from gaining safety planning skills and resources to reduce their risk of exposure to IPV. Multiple factors may have contributed to the positive outcomes of this study. The engagement of the CAAB with diverse stakeholders (e.g., police, IPV service providers, health care providers, KAP NGOs, and women from KAPs with lived experience of IPV) during all phases of adapting and implementing UMAI-WINGS may have reduced barriers to expanding access to key IPV-related services including police protection, legal orders of protection, emergency shelters, counseling, and mental services. The CAAB identified and mobilized a network of IPV, drug and alcohol treatment, harm reduction, mental health, and other services equipped to serve women from KAPs experiencing IPV. The CAAB also worked closely with the study team to identify community-level and organizational-level barriers and facilitators to implementing UMAI-WINGS in partner organizations and make adaptations to improve the implementation infrastructure for delivering UMAI-WINGS in various settings.

Implementation strategies also included training, technical assistance, supervision, and collaborative learning sessions with UMAI-WINGS partner organizations and staff to ensure greater fidelity and uptake of the intervention. UMAI-WINGS embraced core harm reduction principles of empowering women from KAPs to make their own decisions about their relationships and how best to reduce risks and harms from IPV. Further research is needed to identify what multi-level implementation strategies and components of the UMAI-WINGS SBIRT intervention may have contributed to the significant reduction of IPV among women from different KAPs in the intervention community.

The extremely high rates of experiencing all types of IPV in the past 6 months found in this study underscore the major public health and humanitarian crisis that IPV presents for women from KAPs in Kazakhstan. While the 6-month follow-up data demonstrate significant reductions in all forms of IPV, future research should examine whether these effects are sustained over time. Longitudinal follow-up beyond six months is necessary to determine the durability of behavior change, ongoing use of safety planning tools, and continued engagement with services initiated through the UMAI-WINGS intervention. Moreover, the strong associations linking IPV to HIV/STIs, drug overdoses, substance use disorders, PTSD, and other mental health and physical health issues among women from KAPs (El-Bassel et al., 2022; Gilbert et al., 2022, 2015a) further highlight the critical need to redouble the efforts to redress this crisis. The high rate of police abuse experienced by participants in this study, along with the high levels of stigma and discrimination and lack of access to mainstream IPV services, continue to present substantial barriers to ensuring that women from KAPs have the resources and services they need to reduce their risks of experiencing IPV.

The findings of this study highlight the alarmingly high rate of IPV among women from KAPs in Kazakhstan. Given the significant public health concern that IPV represents, it is crucial to incorporate these findings into the development of public policies and programs aimed at reducing IPV and supporting affected women.

One key policy recommendation is the integration of routine IPV screening into health care services, particularly in primary care settings. This would ensure that women at risk of IPV are identified early and provided with the necessary support and interventions. Routine screening could significantly increase the detection of IPV cases that often remain hidden, particularly within marginalized populations.

Additionally, there is a critical need to widen access to IPV services, specifically targeting women from KAPs. This includes expanding access to both government and non-government IPV shelters and the services provided therein. Shelters play an essential role in offering safety, emotional support, and legal assistance, all of which are vital for women seeking to escape abusive situations. Given the unique challenges faced by KAPs in Kazakhstan, it is important that these services be both culturally sensitive and accessible, ensuring that women from these populations can seek help without fear of stigma or discrimination.

These policy recommendations, if implemented, could help to mitigate the pervasive issue of IPV in Kazakhstan and other countries with similar social and cultural contexts. By strengthening the accessibility of IPV services and ensuring early intervention through routine screening, these measures could provide critical support to women in need and contribute to long-term societal change.

Future studies should systematically evaluate the effectiveness of multisectoral partnerships, using implementation science frameworks to identify facilitators and barriers to collaboration across sectors. Research should also explore how engagement structures—such as CAABs—can be optimized to increase community ownership, reduce fragmentation, and improve sustainability of IPV prevention programs. Future implementation trials should prioritize the recruitment of a larger sample of transgender women. This will enable more robust subgroup analyses to assess the differential impact of IPV interventions and ensure that the unique needs of transgender participants are adequately addressed. Future research should continue documenting the specific sociocultural and policy-related barriers encountered by KAPs and how interventions like UMAI-WINGS can be flexibly tailored to overcome them. Such insights would enhance the transferability of the model to other LMIC settings experiencing similar exclusionary policies and service fragmentation.

### 4.1. Limitations of the Study

This study has several limitations that are important to note. First, there may be inextricable differences in the sociodemographics and community-level factors between the intervention and control community that may have contributed to the positive outcomes of this study. Almaty City is primarily urban, with a higher proportion of Russian-identifying participants, while Almaty Oblast is more rural, with a higher proportion of Kazakh-identifying participants. These differences may affect the generalizability of our findings. Given our community-driven multi-level approach to implementing WINGS, a randomized controlled trial with the individual as the unit of analysis was not an appropriate intervention design. Second, this study relied solely on self-reported IPV measures, which may be subject to recall and social desirability bias. Under-reporting is a recognized challenge, particularly in studies involving stigmatized and marginalized groups, such as women from KAPs. While we used validated, standardized tools (e.g., CTS2 and WAST) and computer-assisted self-interviewing (CASI) methods to promote privacy and reduce response bias, these limitations remain. Third, the results of this study have limited generalizability to women from KAPs in Almaty City and Almaty.

### 4.2. Strength and Implications of the Study

These limitations are offset by several strengths, including a robust community engagement approach, a relatively large sample size, controlling for key covariates in the outcome analyses, using widely used standardized measures of IPV, and including women from different KAPs. Taken together, the study findings suggest that a community-driven multi-level approach to adapting and implementing this mHealth IPV SBIRT intervention holds promise for reducing the serious public health threat of IPV among women from different KAPs in Kazakhstan.

## 5. Conclusions

The study findings suggest that a community-based approach to delivering the mHealth UMAI-WINGS intervention was feasible, acceptable, safe, and effective in reducing IPV among women from different KAPs. These results align with previous findings on the WINGS model (Gilbert et al., 2016, 2017, 2015b). UMAI-WINGS, designed as a low-threshold, self-administered mHealth tool, can be successfully implemented and scaled in a wide range of clinical and NGO settings using a community-based approach.

However, future research with a larger-scale, randomized community design and a broader sample of communities is needed to evaluate the outcomes of this community-driven, multi-level approach. Such research may also help identify key mediators and moderators of the IPV SBIRT intervention, which could further optimize UMAI-WINGS delivery across different settings.

The disproportionally high rates of IPV among women from KAPs in this study underscore a call for action for harm reduction policies and programs that expand access to effective services and resources, addressing this widespread epidemic and humanitarian crisis in Kazakhstan.

Although the intervention was adapted for transgender women, and we successfully enrolled 24 participants, the small sample size prevented subgroup analyses. Future research should evaluate UMAI-WINGS across diverse geographic regions and demographic groups within Kazakhstan, including rural, peri-urban, and urban areas. Expanding implementation to additional communities could also assess contextual influences on intervention uptake and outcomes, improving generalizability.

To enhance the validity of IPV outcome data, future studies should incorporate mixed-methods approaches, such as qualitative interviews, ecological momentary assessments, or triangulating reports from service providers and partners, where ethically and logistically feasible. These methods could provide a more nuanced understanding of IPV prevalence and intervention impact. Finally, future research should explore the long-term effects of UMAI-WINGS on IPV outcomes and service engagement, with assessments at 12 months or longer, to better understand the intervention’s sustained effectiveness and scalability.

## Figures and Tables

**Table 1:** Sociodemographic characteristics and key affected population status of the sample (*n* = 458).

	Univariable	Bivariable Comparisons		
	Full Sample *(n* = 458)	Almaty Oblast—Control (*n* = 194)	Almaty City—Intervention (*n* = 264)	*p*-Value
*Age*	36.7 (9.0)	36.1 (9.0)	37.0 (9.0)	0.285
Nationality				
Kazakh	169 (36.9%)	89 (45.9%)	80 (30.3%)	**<0.001**
Russian	142 (31.0%)	38 (19.6%)	104 (39.4%)	
Other	147 (32.1%)	67 (34.5%)	80 (30.3%)	
Education				
9th grade or lower	101 (22.1%)	37 (19.1%)	64 (24.2%)	0.258
Secondary education	145 (31.7%)	57 (29.4%)	88 (33.3%)	
High school	161 (35.2%)	77 (39.7%)	84 (31.8%)	
Bachelor’s or more	51 (11.1%)	23 (11.9%)	28 (10.6%)	
Marital status				
Married	46 (10.0%)	12 (6.2%)	34 (12.9%)	**0.019**
Not married/other	412 (89.96%)	182 (93.8%)	230 (87.1%)	
Homelessness (past year)	186 (40.6%)	104 (53.6%)	82 (31.1%)	**<0.001**
Food insecurity (past year)	254 (55.5%)	133 (68.6%)	121 (45.8%)	**<0.001**
Income (USD)	412.22 (472.34)	412.53 (399.91)	411.99 (519.92)	0.990
Key affected population				
Sex workers	335 (73.1%)	163 (84.0%)	172 (65.2%)	**<0.001**
Persons who use drugs	266 (58.1%)	146 (75.3%)	120 (45.5%)	**<0.001**
Persons living with HIV	140 (30.6%)	34 (17.5%)	106 (40.2%)	**<0.001**

Note: Mean and SD reported for continuous variables. Two-sided *t*-tests utilized for continuous variables. Chi-squared tests utilized for categorical variables. Boldface indicates statistical significance at *p*-value less than 0.05. Demographics reported are from the baseline assessment. Currency exchange rate USD 1 = KZT 520.27 (as of 6 February 2025).

**Table 2:** Descriptives and unadjusted risk ratios of baseline and past 6-month intimate partner violence by intervention group (*n* = 458).

	Univariable	Bivariable Comparisons		
	Full Sample (*n* = 458)	Almaty Oblast—Control (*n* = 194)	Almaty City—Intervention (*n* = 264)	Unadjusted RR (95% CI)
Psychological				
Baseline	296 (64.6%)	127 (65.5%)	169 (64.0%)	0.97 (0.83, 1.15)
Six-month follow-up	256 (55.9%)	146 (75.3%)	110 (41.7%)	**0.56 (0.48, 0.66)**
Sexual				
Baseline	288 (62.9%)	123 (63.4%)	165 (62.5%)	0.98 (0.84, 1.16)
Six-month follow-up	252 (55.0%)	137 (70.6%)	115 (43.6%)	**0.63 (0.54, 0.74)**
Physical				
Baseline	267 (58.3%)	130 (67.0%)	137 (51.9%)	**0.77 (0.66, 0.90)**
Six-month follow-up	211 (46.1%)	143 (73.7%)	68 (25.8%)	**0.30 (0.23, 0.40)**

Note: Boldface indicates statistical significance. Unadjusted RR presented for participants who received intervention vs. those who did not for six-month follow-up.

**Table 3:** Multivariable analyses of intimate partner violence outcomes.

	Multivariable Model	
	aRR (95% CI)	*p*-Value
Past 6-month intimate partner violence		
Psychological	0.77 (0.69, 0.86)	**<0.0001**
Sexual	0.73 (0.63, 0.85)	**<0.0001**
Physical	0.71 (0.63, 0.80)	**<0.0001**

Note: Models adjusted for past 6-month IPV at baseline (for IPV outcomes only), sex worker status, HIV diagnosis, drug use, nationality, marital status, past-year homelessness, and past-year food insecurity. Boldface indicates statistical significance at *p*-value less than 0.05.

**Table 4:** Moderator analyses of intimate partner violence by key population, post-intervention.

	Multivariable Model	
	aRR (95% CI)	*p*-Value
Past 6-month psychological IPV		
Persons living with HIV	1.05 (0.79, 1.40)	0.748
Persons who use drugs	1.23 (0.92, 1.63)	0.160
Sex workers	1.03 (0.78, 1.37)	0.833
Past 6-month sexual IPV		
Persons living with HIV	0.91 (0.71, 1.18)	0.478
Persons who use drugs	1.07 (0.78, 1.48)	0.678
Sex workers	1.06 (0.79, 1.41)	0.697
Past 6-month physical IPV		
Persons living with HIV	1.03 (0.81, 1.32)	0.796
Persons who use drugs	0.95 (0.74, 1.22)	0.690
Sex workers	0.97 (0.74, 1.28)	0.838

Note: Models adjusted for past 6-month IPV at baseline, sex worker status, HIV diagnosis, drug use, nationality, marital status, past-year homelessness, and past-year food insecurity.

## Data Availability

Data can be provided following a reasonable request to the first author.
